# Roles of the second messenger c‐di‐GMP in bacteria: Focusing on the topics of flagellar regulation and *Vibrio* spp.

**DOI:** 10.1111/gtc.12921

**Published:** 2022-01-24

**Authors:** Michio Homma, Seiji Kojima

**Affiliations:** ^1^ Division of Biological Science Graduate School of Science Nagoya University Nagoya Japan

**Keywords:** bacterial flagellum, energy transduction, gene regulation, molecular motor, motility, rotary nano‐machine, signal transduction

## Abstract

Typical second messengers include cyclic AMP (cAMP), cyclic GMP (cGMP), and inositol phosphate. In bacteria, cyclic diguanylate (c‐di‐GMP), which is not used in animals, is widely used as a second messenger for environmental responses. Initially found as a regulator of cellulose synthesis, this small molecule is known to be widely present in bacteria. A wide variety of synthesis and degradation enzymes for c‐di‐GMP exist, and the activities of effector proteins are regulated by changing the cellular c‐di‐GMP concentration in response to the environment. It has been shown well that c‐di‐GMP plays an essential role in pathogenic cycle and is involved in flagellar motility in *Vibrio cholerae*. In this review, we aim to explain the direct or indirect regulatory mechanisms of c‐di‐GMP in bacteria, focusing on the study of c‐di‐GMP in *Vibrio* spp. and in flagella, which are our research subjects.

## Graphical abstract

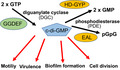

## 1 DISCOVERY OF c‐di‐GMP

Bacteria are known to possess second messengers such as cAMP, cGMP, guanosine pentaphosphate (ppGpp), c‐di‐GMP, and c‐di‐AMP (Hengge, 2020; Opoku‐Temeng et al., 2016). Among these, cAMP was the earliest to be discovered and studied. In *Escherichia coli*, cAMP plays a role in the transcriptional regulation of glucose metabolism; on the other hand, in animals, it is involved in a great variety of regulatory activities in conjunction with G protein‐coupled receptors (GPCRs) (Gancedo, 2013). c‐di‐GMP is involved in a variety of environmental responses of bacteria.

In animals, cyclic nucleotides such as cAMP and cGMP are known to be involved in various biological functions. Recently, bis (3',5')‐cyclic diguanylic acid (c‐di‐GMP) has been shown to be an allosteric activator that regulates various reactions during bacterial adaptation to the environment. c‐di‐GMP is widely produced in the bacterial kingdom and is unique to this kingdom. c‐di‐GMP was found to regulate enzyme production, where it acts as a positive regulator of cellulose synthase in *Gluconacetobacter xylinus*, a bacterium known to synthesize cellulose, and was found to bind to allosteric active factors of the membrane‐bound cellulose synthesis system (Ross et al., 1985, 1987; Weinhouse et al., 1997). It has also been shown to regulate the synthesis and function of the extracellular components of flagella and cilia and the synthesis of exopolysaccharides (Romling & Amikam, 2006). c‐di‐GMP exerts a variety of functions through various binding proteins that have a PilZ domain. PilZ is a small cytoplasmic protein of 13 kDa expressed as part of the pili‐producing operon and is essential for pili biosynthesis. Based on genome analysis, the PilZ domain is predicted to be the leading site for c‐di‐GMP binding (Amikam & Galperin, 2006). The binding of c‐di‐GMP to the PilZ domain was demonstrated using purified PilZ domain proteins (Ryjenkov et al., 2006), and the crystal structure of the c‐di‐GMP‐bound PilZ domain has also been reported (Benach et al., 2007).

## 2 c‐di‐GMP SYNTHESIS AND DEGRADATION ENZYMES IN BACTERIA

**FIGURE 1:**
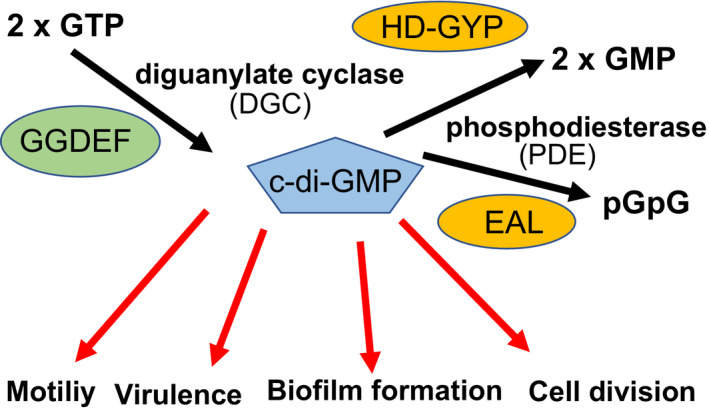
Model of c‐di‐GMP signaling. The concentration of c‐di‐GMP is regulated by a synthase of diguanylate cyclase (DGC) and a degrading enzyme of phosphodiesterase (PDE). The binding of c‐di‐GMP to various effector molecules regulates various functions, motility, virulence, biofilm formation, cell division, among other functions

c‐di‐GMP is synthesized from two molecules of GTP by diguanylate cyclase (DGC) and degraded to two molecules of GTP by phosphodiesterase (PDE). Cyclase activity is encoded in the GGDEF domain (also known as DUF1) (Paul et al., 2004; Ryjenkov et al., 2005). The GGDEF domain of DGC was named from the five amino acid residues GGDEF of PleD, which controls flagellar formation in *Caulobacter crescentus* (Hecht & Newton, 1995). Later, it was shown that this motif forms the active center of the DGC (Schirmer & Jenal, 2009). Phosphodiesterase activity is encoded by the EAL (DUF2) domain (Christen et al., 2005; Schmidt et al., 2005; Tamayo et al., 2005) and the HD‐GYP domain (Ryan et al., 2006) (Figure 1).

Genome analysis of various bacteria shows that many of the GGDEF, EAL, and HD‐GYP domain proteins, which are thought to be responsible for the synthesis and degradation of c‐di‐GMP, are encoded in the genome (Chou & Galperin, 2016). In *Vibrio cholerae*, there are 31 proteins with GGDEF, 11 proteins with both GGDEF and EAL domains, 12 proteins with EAL, and 9 proteins with HD‐GYP domains; thus, a total of 62 proteins involved in the synthesis and degradation of c‐di‐GMP were inferred in the bacterial genome. As shown in Table 1, the number of c‐di‐GMP synthesis and degradation proteins in the *Vibrio* spp is much more than that in *E. coli*. However, there are no homologs of these factors in archaea kingdom. Although it is not yet clear why such a large number of c‐di‐GMP synthetases are required, it is obvious that they evolved during ancient times to help organisms to cope with the harsh environment of Earth. It should be noted that most of those predicted c‐di‐GMP synthesis/degradation proteins are uncharacterized, and so they might exert other functions rather than the c‐di‐GMP biogenesis.

**TABLE 1:** Number of GGDEF, EAL, HD‐GYP, and PilZ domain proteins in the genomes

	Proteins	GGDEF	GGDEF+EAL	EAL	HD‐GYP	PilZ	MshEN	Other
1:*E. coli*	4,132	12	7	10	–	2	1	BcsE
2:*P. aeru*	5,567	17	16	5	3	8	2	FleQ
3:*V. chol*	3,835	31	10	12	9	5	1	VpsT, FleQ
4:*V. harv*	6,055	32	12	14	5	5	1	VpsT, FleQ
5:*V. para*	4,832	28	16	13	5	5	1	VpsT, FleQ
6:*V. anti*	4,518	29	16	11	4	5	1	VpsT, FleQ

Note

The data were obtained from the Web site (http://ncbi.nlm.nih.gov/Complete_Genomes/c‐di‐GMP.html). 1: *E. coli*, 2: *P*. *areruginosa*, 3: *V. cholerae*, 4: *V*. *harveyi*, 5: *V. parahaemolyticus*, 6: *V*. *antiquaries*.

## 3 STRUCTURE OF c‐DI‐GMP‐BINDING PROTEIN

**FIGURE 2:**
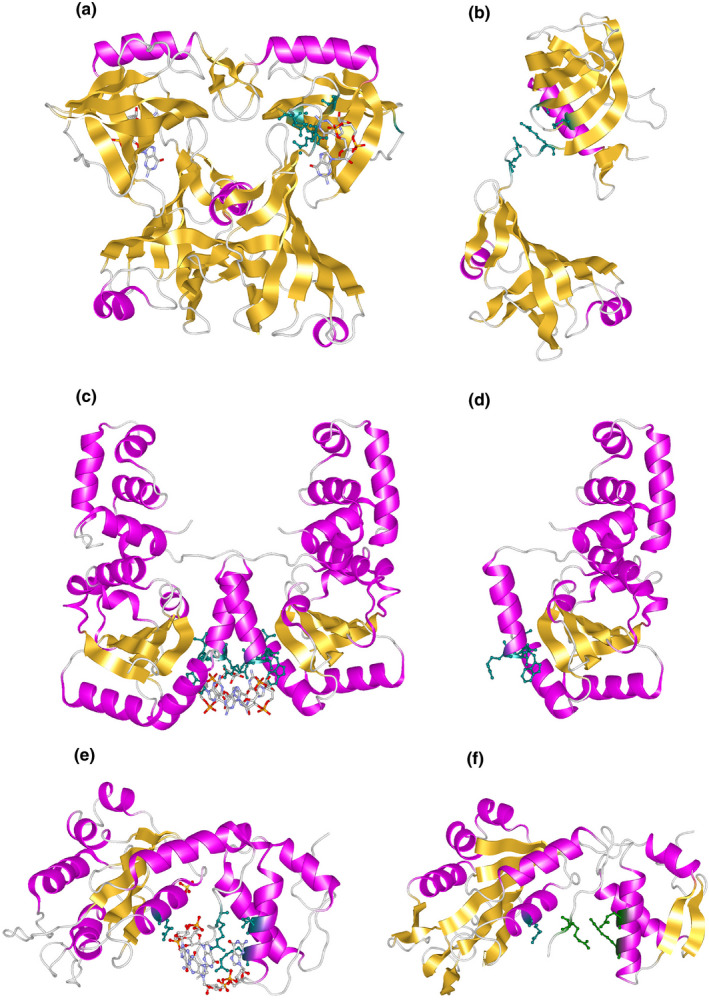
Structures of c‐di‐GMP‐binding proteins. Crystal structure models of c‐di‐GMP‐binding PlzD (a), c‐di‐GMP‐free PlzD (b), c‐di‐GMP‐binding VpsT (c), c‐di‐GMP‐free VpsT (d), c‐di‐GMP‐binding FleQ (e), and c‐di‐GMP‐free FleQ (f). The c‐di‐GMP‐binding residues are shown in the helix model with the stick model of the residues in dark green. c‐di‐GMP is shown in the stick model

Among the various c‐di‐GMP‐binding proteins, the structure of *V. cholerae* PilZ domain‐containing protein D (PlzD) was determined first (Benach et al., 2007). PlzD is one of the five PilZ domain proteins that was identified from the *V. cholerae* genome sequence as VC0042 protein. The N‐terminal domain of PlzD is homologous to that of YcgR. YcgR is involved in flagellar gene regulation in *E. coli* and has a PilZ domain at its C‐terminus. It was later shown to bind c‐di‐GMP and act on a flagellar motor (Hou et al., 2020). *ycgR* was initially identified as a suppressor gene for the defect of the nucleoid protein H‐NS, which causes the repression of flagellar gene expression. Transposon insertion in *ycgR* suppresses the H‐NS defect and restores flagellar formation (Ko & Park, 2000). PlzD has a c‐di‐GMP binding consensus sequence of RxxxR and D/NxSxxG in the PilZ domain on the C‐terminal side region. Benach et al. (2007) determined the apo structure without c‐di‐GMP and the complex structure in which one molecule of c‐di‐GMP was bound to a PilZ domain, revealing that c‐di‐GMP‐bound PlzD formed a dimer. Notably, the binding of c‐di‐GMP to the PilZ domain causes a large structural change (Figure 2a,b). In *E. coli* YcgR, the C‐terminal PilZ domain interacts with the flagellar stator protein MotA in the motor, and the N‐terminal domain interacts with other motor proteins (assuming the rotor protein FliG). Thus, it is currently thought to inhibit motor function (Hou et al., 2020). Recently, it has been found that degradation of the σ^S^ sigma factor by ClpXP protease occurs in a c‐di‐GMP‐dependent manner and affects flagellar gene control (Nieto et al., 2020). However, it is not known whether YcgR directly affects flagellar gene regulation.

Among *Vibrio* spp., VpsT is a c‐di‐GMP‐binding protein whose structure has been determined (Krasteva et al., 2010). As described in Section 5, this protein belongs to the LuxR and CsgD families. It is a two‐component regulatory effector protein that is phosphorylated. Although the putative phosphorylation site is conserved, the other residues involved in phosphate transfer are not conserved. The N‐terminus contains the receiver (REC) domain, which contains the transcriptional control domain with a helix‐turn‐helix (HTH) motif and a c‐di‐GMP conserved sequence of four residues (W[F/L/M][T/S]R). The binding molar ratio of c‐di‐GMP to VpsT is 1:1, and two molecules of c‐di‐GMP bind to the dimer of VpsT. However, it seems that c‐di‐GMP is not necessary for VpsT dimerization. The binding of c‐di‐GMP changes the structure of the HTH domain at the DNA‐binding site, thus changing its interaction with DNA and affecting the transcriptional activity (Figure 2c,d).

Another well studied structure among the c‐di‐GMP‐binding proteins is that of the FleQ protein of *Pseudomonas aeruginosa*. As described in Section 7, FleQ is an ortholog of FlrA, known as the flagellar gene master regulator of *V. cholerae*, and functions as a flagellar gene master regulator in *P. aeruginosa* (Baraquet & Harwood, 2013; Hickman & Harwood, 2008). FleQ has a REC receiver domain at the N‐terminus, a bacterial AAA+ATPase/σ54 interaction domain at the center, and a HTH DNA‐binding domain, which is highly homologous to the NtrC subfamily, a family of bacterial enhancer‐binding proteins (bEBP). However, it is speculated that the control mechanism of NtrC and FleQ is different. FleQ is thought to form a stable hexamer ring structure and suppress transcription when c‐di‐GMP is not bound. As the c‐di‐GMP concentration rises and c‐di‐GMP binds to FleQ, the hexamer is deformed. The structure then undergoes conformational changes that allow the transcription of flagellar genes. The control region that binds to c‐di‐GMP exists at the interface between the REC and AAA+domains. The structural data show that c‐di‐GMP binds to the LFR^144^S motif, the R185 and N186 residues of the post‐Walker A region, and the ExxxR^334^ sequence (Figure 2e,f) (Matsuyama et al., 2016). A recent report suggested that c‐di‐GMP and FleQ regulate cAMP by modulating *cyaA* expression (Xiao et al., 2021).

## 4 REGULATION OF VIRULENCE FACTORS BY c‐di‐GMP IN *VIBRIO* spp.

**FIGURE 3:**
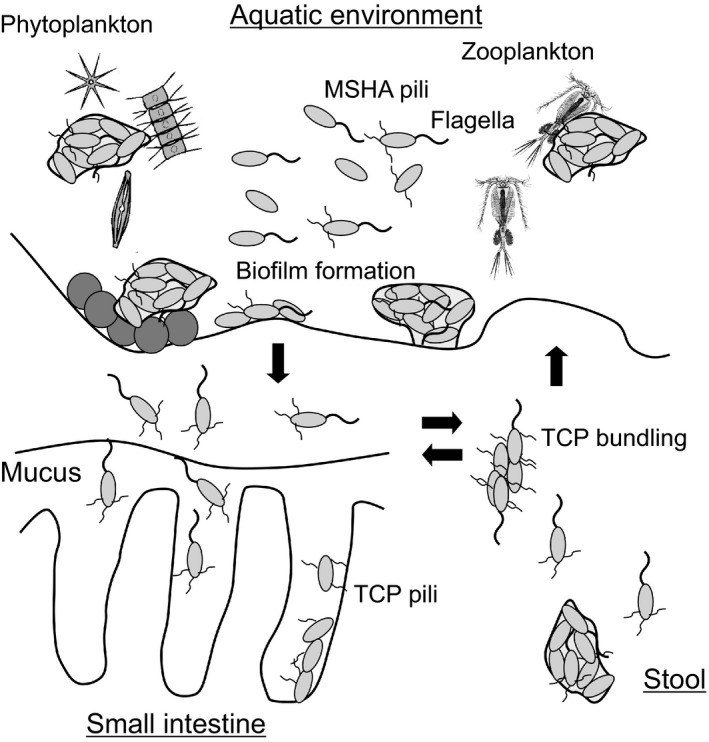
Biofilm formation during the life cycle of *Vibrio cholerae*. In the aquatic environment, *V. cholerae* is present in two states: a highly motile planktonic state and an attached state on zooplankton, phytoplankton, carcasses, and sediment surfaces, the latter of which eventually forms a biofilm. Type IV pili, mannose‐sensitive hemagglutinin (MSHA) pili, and flagella contribute to the initial attachment to living and non‐living surfaces. Then, cells produce an extracellular matrix and polysaccharides that form mature biofilms. It is not clear whether there is loss of flagella and pili during biofilm formation. However, it has been reported that flagella are shed in the stationary phase. *V. cholerae* can infect humans from this aquatic environment and cause seasonal epidemics. When the bacteria colonize the intestine, aggregates of floating cells and biofilm‐forming cells producing TCP pili have been observed. These are expelled as stool and can reinfect new humans or return to the aquatic environment. The figure was created based on a previous study by Teschler et al. (2015)

**FIGURE 4:**
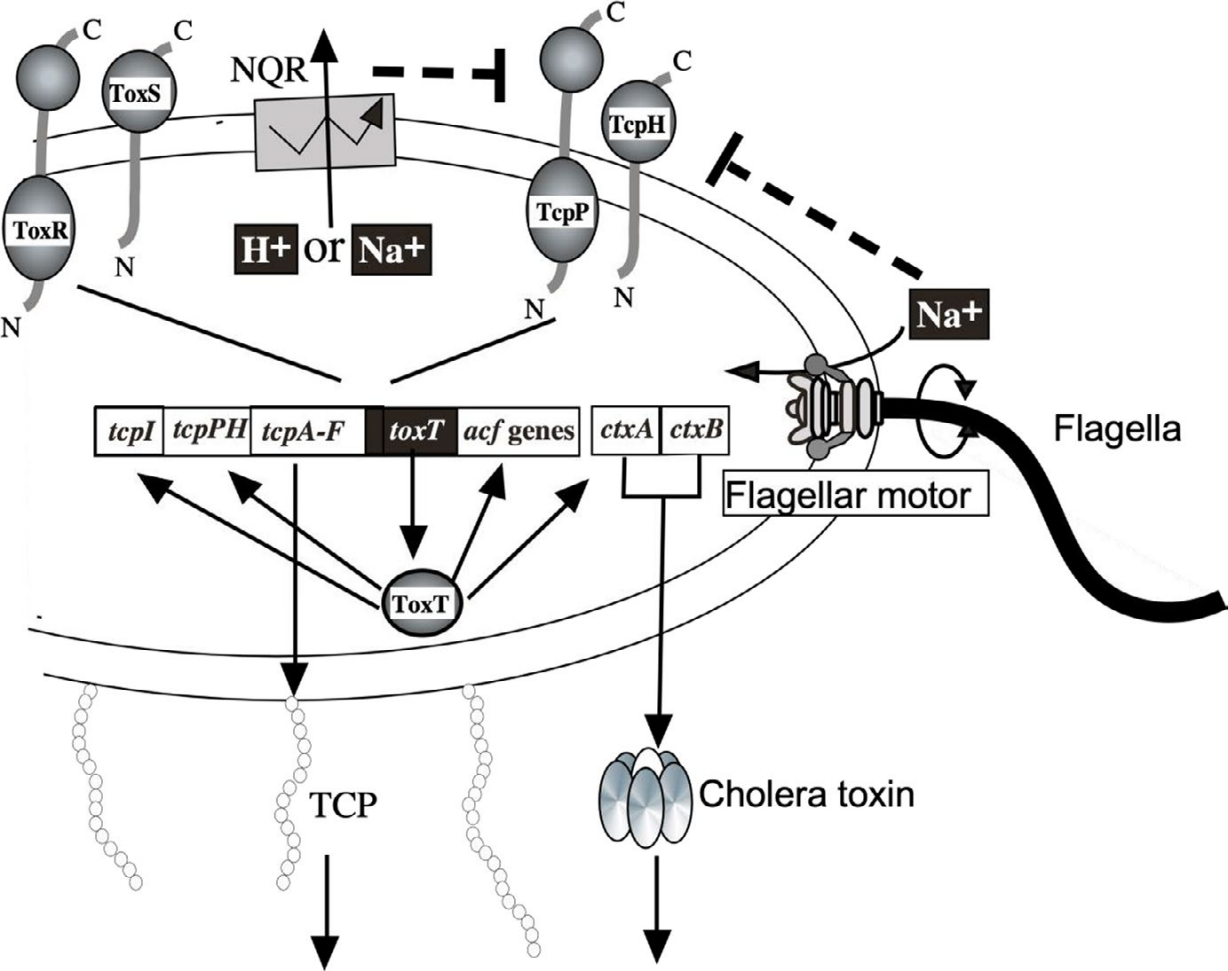
Regulatory model of *V. cholerae* virulence factors. The membrane proteins TcpH/TcpP and ToxS/ToxR receive external environmental stimuli and positively regulate the transcription of *toxT* gene. The synthesized ToxT acts as a transcription factor and regulates the transcription of downstream virulence factors. The movement of sodium ions through the sodium‐driven flagellum and respiratory chain seems to affect the transcription of *toxT*. Although not shown in the figure, there is a pathway in which the quorum signal is accepted by the sensor kinases CqsS and LuxQ, and influences the production of virulence factors by a multi‐step signaling cascade through LuxO. The involvement of these very complex genes controls the regulation of the expression of virulence factors such as cholera toxin and TCP. The figure was created based on previous studies (Krukonis et al., 2000; Ramamurthy et al., 2020; Zhu et al., 2002)

The life cycle of *V. cholerae*, the pathogenic bacterium causing cholera, can be divided into two main phases: the free‐swimming phase and the virulent phase. During the former phase, *V. cholerae* is mainly found outside the host. It does not express virulence factors and actively moves around. In the environment, the bacteria grow either in a swimming form, using flagella to move actively in the water, or as an adherent form, forming biofilms on the surface of fish, shrimp, plankton, sediment, and so on (Figure 3) (Teschler et al., 2015). In order to form biofilms in the aquatic environment, they attach to the surface of aquatic organisms through the formation of mannose‐sensitive hemagglutinin (MSHA) pili which are type IV pili. On entering the host and reaching the surface of the infected intestine, they shift to the latter state, where they lose motility; at the same time, they form toxin co‐regulated pilus (TCP), which is required for colonization of the small intestine, as well as virulence factors such as cholerae toxin (CT), which causes electrolyte secretion (leakage) (Figure 4) and the characteristic cholera symptom of diarrhea.

Several flagellar genes and chemotaxis‐related genes are known to be involved in *V. cholerae* pathogenicity. Transcription factors, including AphA/B, TcpP/H, ToxR/S, and ToxT, have been shown to regulate the expression of CT and TCP (Reidl & Klose, 2002). ToxT plays a major role in expressing virulence factors in *V. cholerae* and is a transcriptional activator that activates the promoter of *ctxAB*, which encodes CT, resulting in toxin production and pathogenicity (Champion et al., 1997; Skorupski & Taylor, 1997). ToxR and TcpP activate the expression of *toxT* (both inner membrane proteins with a DNA‐binding domain in the cytoplasmic region) (Häse & Mekalanos, 1998). The induction of these genes is influenced by the external environment, such as temperature, pH, and sodium ion concentration. In addition to CT, ToxT activates the transcriptions of the *tcp* (toxin co‐regulated pilus) gene cluster, including *tcpA*, which encodes the main component of TCP, as well as the *acf* (accessory colonizing factor) gene cluster (Brown & Taylor, 1995; Faruque et al., 1998). The expression of many virulence factors, such as CT (cholera toxin) and TCP pili, is coordinately regulated by the transcriptional regulator ToxR and its downstream ToxT (Figure 4). It has been found that the expression of virulence factors such as CT, TCP pili, hemolysins, and biofilms is upregulated in *V. cholerae* with loss of motility. In contrast, the expression of these virulence factors is downregulated in mutant strains with increased motility (Gardel & Mekalanos, 1996).

The above evidence suggests a common factor that controls the expression of flagellar proteins and virulence factors. In response to various physiological conditions, alternative σ factors that regulate the transcription of various genes in bacteria were considered. The σ factor used in normal growth is called σ^70^ because of its molecular weight of 70 kDa. The alternative σ‐factor, σ^54^, regulates the expression of nitrogen assimilation genes, flagellar genes, and pili genes (Kustu et al., 1989). σ^54^‐deficient strains of *V. cholerae* were generated, and the expression of virulence factors was examined (Klose & Mekalanos, 1998). The production of CT and TCP pili was not affected. However, the flagellar formation was defective, and the mutation affected the expression of glutamine synthetase. In addition, colony formation ability of the σ^54^‐deficient strain of *V. cholerae* in an infant mouse model system was abolished. It was suggested that this defect in colony formation was not derived from the indirect effects of flagellar defects and glutamine synthetase levels. However, how environmental signals regulate the sigma factor still remains unclear (Khan et al., 2020).

c‐di‐GMP appears to be a factor that links the expression of these pathogenic factors. Although there is no direct evidence that c‐di‐GMP is involved in pathogenicity, it is known that proteins with GGDEF and EAL domains are involved in biofilm formation in *Vibrio* spp. (Boles & McCarter, 2002; Bomchil et al., 2003; Rashid et al., 2003). In this context, it was revealed that VieA, which is involved in the production of cholera toxin by the two‐component regulatory system of *Vibrio*, negatively regulates the expression of the *Vibrio* polysaccharide (*vps*) genes, which are required for biofilm formation (Tischler & Camilli, 2004). In addition, it was found that VieA has an EAL domain, and its degradation activity regulates the concentration of c‐di‐GMP, which in turn regulates gene expression (Dey et al., 2013; Tamayo et al., 2005).

## 5 BIOFILM FORMATION AND c‐di‐GMP IN *VIBRIO* spp.

**FIGURE 5:**
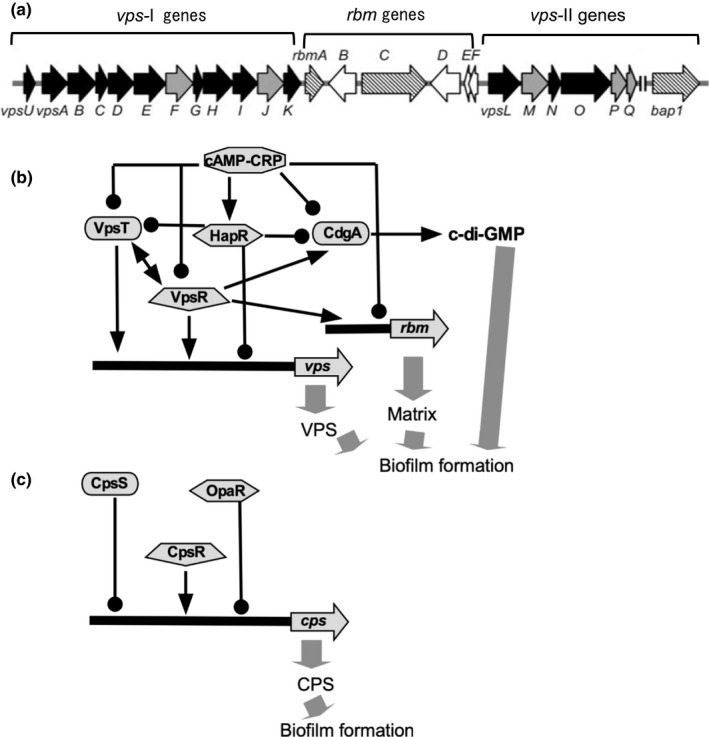
Gene regulation of biofilm formation. (a) Biofilm‐forming genes of *V. cholerae*. *rbm* gene cluster encoding matrix proteins are located between *vps*‐I and *vps*‐II clusters of genes (Fong et al., 2010). (b) Regulation of biofilm formation in *V. cholerae*. VpsT and VpsR are the major direct transcriptional regulators of polysaccharide synthesis, required for biofilm formation (Teschler et al., 2015). (c) Regulation of biofilm formation in *V. parahaemolyticus*. Transcription factors homologous to the *V. cholerae* regulatory proteins are involved, but the regulatory mechanism is slightly different (Yildiz & Visick, 2009). The arrow ends represent positive regulation, and the black circle ends represent negative regulation

Biofilm formation occurs when *V. cholerae* changes from the swimming state using flagella to the adherent state using pili. The main constituents of biofilms are the extracellular polysaccharide (VPS), and the vps‐I (*vpsU*, *vpsA*‐*K*, VC0916‐27) and vps‐II (*vpsL*‐*Q*, VC0934‐39) operons encode the genes required for VPS production. In addition to VPS, matrix proteins (RbmA, RbmC, and Bap1) are necessary for biofilm formation. The genes required for matrix protein synthesis are clustered between the vps‐I and vps‐II regions (Figure 5a) (Fong et al., 2010). The *vps* gene expression is regulated by VpsR, VpsT, or HapR (Fong & Yildiz, 2008) and other factors (Cheng et al., 2018; Teschler et al., 2017). VpsR is a σ^54^‐dependent transcriptional regulator that contains an AAA‐type ATPase domain and a DNA‐binding domain. HapR is a member of the TetR family that binds to DNA and represses transcription. As mentioned in Section 3, VpsT is a protein composed of an N‐terminal receiver (REC) domain and a C‐terminal helix‐turn‐helix (HTH) domain that mediates DNA binding and is a phosphorylated effector protein of the LuxR and CsgD families of two‐component regulatory systems. The dimerization of VpsT on binding of c‐di‐GMP has a more substantial effect on transcription than phosphorylation by upstream kinases (Krasteva et al., 2010). It has been found that VpsR regulates the expression of another transcription factor, TfoY, which is regulated in a c‐di‐GMP‐dependent manner (Pursley et al., 2018). Using an in vitro system, it has been shown that VpsR acts on the promoters of the *vps* genes to regulate their expression depending on σ^70^ RNA polymerase and c‐di‐GMP (Hsieh et al., 2018). VpsR also regulates the production of matrix proteins and further regulates the expression of genes (*eps* genes), which are required for the secretion of proteins of the type II secretion apparatus out of the bacterium in a c‐di‐GMP‐dependent manner (Sloup et al., 2017). The promoter regions of the *eps* genes have been shown to have two promoters, σ^E^ and σ^70^ dependent ones (Zielke et al., 2014). Furthermore, cAMP is also involved in biofilm formation, depending on the concentration of c‐di‐GMP.

As described above, biofilm formation is regulated in a very complex manner in *V. cholerae*. In *V. parahaemolyticus*, a well‐known pathogenic bacterium of *Vibrio*, the major component of biofilms is the extracellular polysaccharide, and the genes required for its synthesis are named *cps* (capsular polysaccharide) and are clustered together at one locus (Yildiz & Visick, 2009). The region contains the genes necessary to synthesize polysaccharides, and the composition of genes is slightly different from that of *V. cholerae*. The composition of the VPS of *V. cholerae* is glucose (52.6%), galactose (37.0%), N‐acetylglucosamine (5.1%), mannose (3.8%), and xylose (1.5%), and that of *V. parahaemolyticus* is glucose (34.6%), galactose (27.8%), fucose (21.3%), N‐acetylglucosamine (13.9%), arabinose (1.0%), mannose (0.7%), and N‐acetylgalactosamine (0.6%) (Enos‐Berlage & McCarter, 2000; Yildiz & Schoolnik, 1999). In terms of gene regulation, similar transcriptional regulators (OpaR, CpsR, CpsS) are involved in *V. cholerae* and *V. parahaemolyticus*. However, the regulatory mechanisms seem to be slightly different among the species (Yildiz & Visick, 2009) (Figure 5c). OpaR, which is a homologue of LuxR in *V. harvery* (McCarter, 1998), is also recognized as the master regulator of quorum sensing, and approximately 11 transcription factors controlled by OpaR have been identified (Kernell Burke et al., 2015). OpaR also regulates the c‐di‐GMP concentration (Zhang et al., 2021). It seems that very complicated regulation is present in the different *Vibrio* species.

## 6 TWO TYPES OF FLAGELLA IN *VIBRIO* spp.

*V. parahaemolyticus* and its close relative, *V. alginolyticus*, have two functionally distinct types of flagella: a single polar flagellum and multiple lateral flagella (McCarter, 2004). *V. cholerae* has one polar flagellum, whereas *V. fischeri* has multiple polar flagella. The energy source of flagellar motility in the polar and lateral flagella was investigated, and it was found that Na^+^ motive force drives the polar flagellum, whereas H^+^ motive force drives the lateral flagellum (Atsumi et al., 1992; Kawagishi et al., 1995). The polar flagellum is expressed constitutively, whereas the lateral flagellum is expressed under highly viscous conditions, suggesting that *Vibrio* spp. senses an increase in viscosity. Experiments using phenamil (a derivative of amiloride, a sodium channel inhibitor), an inhibitor of the Na^+^‐driven motor of the polar flagellum, showed a correlation between decreased polar flagellar rotation and expression of the lateral flagellar genes. This suggests that polar flagella act as mechanical sensors and regulate the expression of lateral flagellar genes (Kawagishi et al., 1996).

Although this phenomenon was discovered more than 30 years ago, it is still unclear how the polar flagellum senses viscosity. For *V. cholerae*, it has been reported that polar flagellar movement influences the control of biofilm formation. Lack of flagellar filament protein increases the concentration of c‐di‐GMP in cells, and loss of MotY, which is essential for the motor function of the polar flagellum, suppresses the increase in c‐di‐GMP (Wu et al., 2020). The mechanism by which the polar flagellar gene regulates c‐di‐GMP levels remains unclear. In *V. parahaemolyticus*, ScrABC has been reported to regulate the expression of the lateral flagellum and biofilm formation (Boles & McCarter, 2002). ScrC is a membrane protein with a cytoplasmic region containing a GGDEF‐EAL domain, has c‐di‐GMP degradation and synthesis activities, and regulates c‐di‐GMP levels upon signaling (Trimble & McCarter, 2011). The S signal, a pheromone signal that helps communicate between cells in co‐culture to regulate surface colonization, is produced by the pyridoxal‐phosphate‐dependent aminotransferase of ScrA and is sensed by SscL (VPA1462) and SscS (VPA1492), homologous methyl‐accepting chemotaxis proteins (Lamb et al., 2019). It has been shown that SscS is expressed as part of the lateral flagellar gene system, with the scrABC operon responsible for S signal production (Gode‐Potratz et al., 2011). The genes, *scrJ* (VPA1115), *scrL* (VPA1069), and *lafV* (VPA1547), which encode proteins that contribute to the Scr network, have been identified (Kimbrough & McCarter, 2021). ScrJ and ScrL are tetratricopeptide repeat (TPR)‐coupled GGDEF proteins. LafV, which is encoded by the last gene in the lateral flagellar operon and contains a degenerate phosphodiesterase (EAL) domain, represses the transcription of multiple genes in the surface sensing regulon in the presence of LafK, the primary swarming regulator.

**FIGURE 6:**
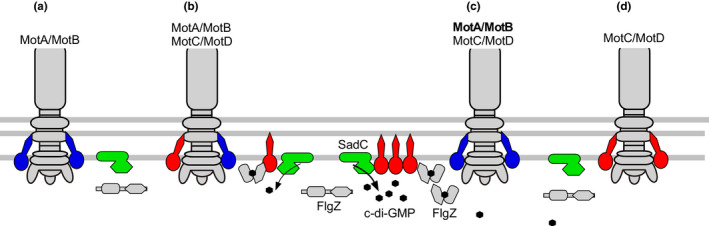
Model for the involvement of flagellar stators in c‐di‐GMP levels. Interactions between the DGC membrane protein SadC (green) and the stator complex in the *ΔmotCD* mutant strain (a), the wild‐type strain (b), the strain expressing MotAB from a multicopy plasmid (c), and the *ΔmotAB* mutant strain (d). The MotA/MotB stator complexes are shown in blue, and the MotC/MotD stator complexes are shown in red. The effector FlgZ can interact with MotC when in the c‐di‐GMP bound state. The figure was adapted from a previously published paper (Baker et al., 2019)

Recently, a study of *P. aeruginosa* provided a clue to the mechanism of c‐di‐GMP regulation in the flagellar system (Baker et al., 2019). *P. aeruginosa* has two types of flagellar stator complexes, MotA/MotB and MotC/MotD. The stator of MotC/MotD enables the flagellum to perform migratory movements such as crawling on the surface under highly viscous conditions. It has been shown that FlgZ, which has a PilZ domain and is homologous to *E. coli* YcgR, affects this migratory movement and interacts with MotC (Baker et al., 2016). When MotC/MotD was knocked down, the c‐di‐GMP levels decreased; on the other hand, when MotC/D was overexpressed, the c‐di‐GMP levels increased and biofilm formation was induced. It has also been found that MotC acts on the transmembrane region of SadC, a membrane protein with a GGDEF motif in its C‐terminal cytoplasmic region (Baker et al., 2019). Based on this evidence, a model was presented in which the MotC/MotD stator complex interacts with SadC, a DGC, to increase the c‐di‐GMP synthesis activity (Figure 6). It is likely that the PomA/PomB stator complex of *Vibrio* spp. may interact with a membrane protein with c‐di‐GMP degradation and synthesis activity to alter c‐di‐GMP concentrations.

## 7 FLAGELLAR FORMATION IN *VIBRIO* spp.

As mentioned above, flagella motility is not necessary for the growth of *Vibrio* spp. under nutrient‐rich culture conditions. In the laboratory environment, flagellum‐deficient strains grow exactly as wild‐type strains. The cost of flagellar maintenance is very high. In *Shigella*, which causes dysentery and is very closely related to *E. coli* or *Salmonella*, the flagellar genes are thought to have been lost as they are no longer needed (Tominaga et al., 2005). The flagellum is composed of more than 20 structural proteins, and more than 50 genes encode and regulate the expression of these flagellar structural proteins. To minimize the maintenance cost, flagellar gene expression is tightly regulated when not needed (Chevance & Hughes, 2008; Terashima et al., 2008).

**FIGURE 7:**
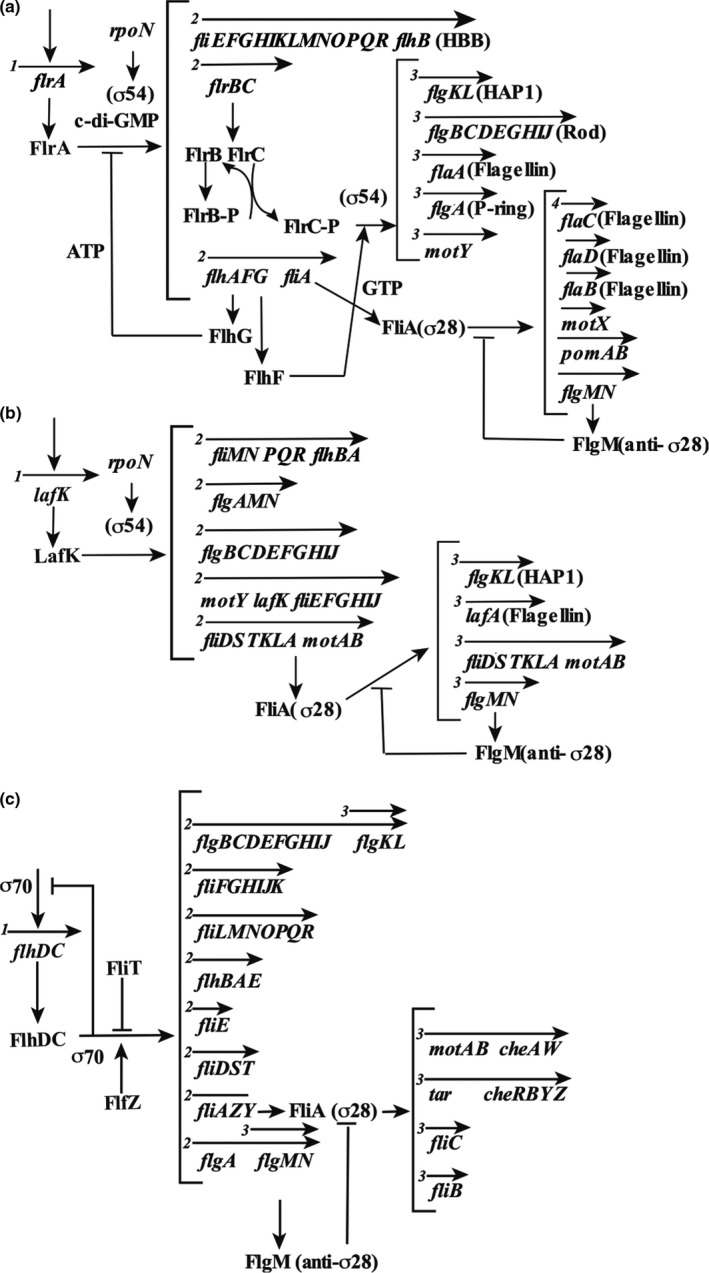
Flagellar genes. The genes of the sodium ion‐driven polar flagella of *V. cholerae* (a), the proton‐driven lateral flagella of *V. parahaemolyticus* (b), and the proton‐driven peritrichous flagella of *E. coli* (c) and their gene configurations are shown. The arrows above the gene names indicate the transcription units, and the numbers at the beginning indicate the order of the transcription hierarchy

In *V. parahaemolyticus* and *V. cholerae*, flagellar gene expression regulation of polar flagellar genes has been postulated (Echazarreta & Klose, 2019; McCarter, 2004; Syed et al., 2009). In *V. cholerae*, the transcriptional regulation of the operon was modeled by dividing the operon into four classes according to the order of expression (Figure 7a) (Correa et al., 2000; Prouty et al., 2001). Motor genes such as *motAB* and *motY*, and the flagellin‐coding genes *flaB*, *flaC*, *flaD*, *flaE*, and the *flgM* operon which encodes a putative anti‐σ factor, have σ^28^‐type promoter sequences and are expressed depending on σ^28^ encoded by the *fliA* gene. In contrast, *flaA*, which encodes core flagellin; *motX*, which encodes a motor protein; and structural genes of the hook, basal flagellar bodies, or hook‐associated proteins (HAPs) are expressed depending on FlrC and σ^54^. The *flrA* gene is transcribed first, and its product, FlrA, causes transcription of the class II operon with σ^54^. The phosphate transfer from FlrB to FlrC activates FlrC in the two‐component system. The binding of c‐di‐GMP to FlrA has been reported to inhibit the binding of FlrA to the *flrBC* operon, thus preventing transcription and flagellar formation (Srivastava et al., 2013). Similar regulation of flagellar gene expression by c‐di‐GMP has been observed in *P. aeruginosa*, and the function of FleQ, a homolog of FlrA with approximately 50% homology, has been well studied. FleQ is classified as an AAA+‐type ATPase and a master regulator of the polar flagellar genes in *P. aeruginosa*, as described in Section 3.

The polar flagellar genes of *V. parahaemolyticus* are also regulated by transcriptional control similar to that of *V. cholerae* (although the genes are named differently) (Kim & McCarter, 2000; McCarter, 2001). Transcriptional regulation of the lateral flagellar genes in *V. parahaemolyticus* was also analyzed and found to be hierarchically regulated (Figure 7b). Interestingly, the master regulator of the lateral flagellar genes in *V. parahaemolyticus* is the σ^54^ type. However, the downstream regulator is similar to the σ^28^ type of *E. coli* (Figure 7c) (Stewart & McCarter, 2003). In *V. campbellii*, a marine bacterium belonging to the harveyi clade that shares similar free‐living and pathogenic life cycles to other members of the *Vibrionaceae* (Urbanczyk et al., 2013), the flagellar genes are involved in the pathogenesis of infection caused by this microorganism, and inhibition of motility significantly decreases host mortality during infection (Petersen et al., 2021; Yang & Defoirdt, 2015). While *V. campbellii* encodes the genes for flagella and chemotaxis, and these genes are conserved in other *Vibrio* species, the expression and function of these genes have not been characterized. In addition, the *V. campbellii* genome encodes genes that are homologous to *V. parahaemolyticus* lateral flagellar genes for swarming motility. However, only a few strains express these genes with little phenotypic or genetic characterization (Allen & Baumann, 1971; Shinoda et al., 1992). It has been shown that the regulatory hierarchy of *V. campbellii* transcription of the flagellar and chemotaxis genes is similar to that of other *Vibrio* spp.; however, some differences have been observed in the roles of σ^54^‐dependent regulators (Petersen et al., 2021). These differences might have been adapted to the living environment of *Vibrio* spp.

## 8 THE ACCELERATOR AND BRAKE OF FLAGELLAR MOTOR ROTATION INFLUENCED BY c‐di‐GMP

When the growth environment suddenly changes, the load applied to the bacterium changes significantly, which affects the migration pathways. To respond to such changes in the external environment, the ability to adjust the flagellar motor's rotation speed that controls the bacterium's movement is very important. For example, the motor needs to generate more torque (accelerate) to drive the rotor under a heavy load. Indeed, recent studies have shown that cells respond to upshifts in load by increasing the number of stator units surrounding the rotor (Lele et al., 2013; Pourjaberi et al., 2017; Terahara et al., 2017; Tipping et al., 2013). Approximately 10 stator units are incorporated to generate high torque at high loads. Few stator units are assembled to rotate the motor around the rotor at low loads. Therefore, the stator unit acts as a load sensor and adjusts the rotation speed to external load changes. The speed of the motor in response to environmental changes is also adjusted when the physiological state of the cell changes from a swimming state to an adherent state during biofilm formation (Baker & O'Toole, 2017). High levels of intracellular c‐di‐GMP have been shown to promote surface attachment and biofilm formation (Hengge, 2009; Jenal et al., 2017).

**FIGURE 8:**
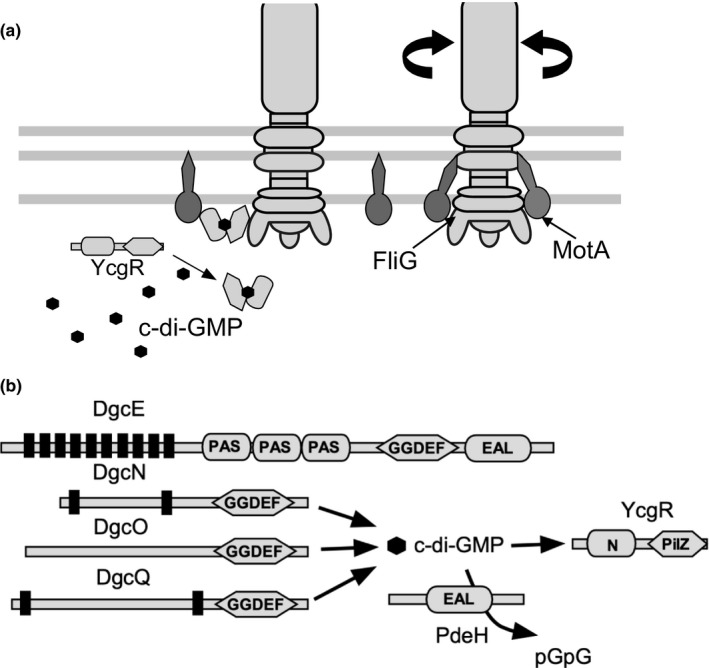
Model for inhibition of the motor rotation by c‐di‐GMP‐binding protein. The concentration of c‐di‐GMP is altered by the synthesis and degradation of various enzymes in *E. coli*, and the binding of c‐di‐GMP to YcgR inhibits the motor (Boehm et al., 2010). (a) Flagellar basal body structure and periplasmic stator units (MotA/MotB) spanning the envelope (inner and outer membranes) of *E. coli*. YcgR, a c‐di‐GMP‐binding protein, interacts with the stator and rotor in a c‐di‐GMP‐dependent manner. (b) c‐di‐GMP is synthesized by four different DGCs (DgcE, DgcN, DgcO, and DgcQ) and hydrolyzed by the PDE, PdeH, to linear di‐GMP (pGpG)

In *E. coli*, it has been reported that deletion of the DGC‐encoding gene, *yhjH*, and increasing the concentration of intracellular c‐di‐GMP decreases the swimming speed of the bacteria, while deletion of a PDE‐synthesizing gene and decreasing the concentration of c‐di‐GMP increases the swimming speed (Ko & Park, 2000). The inhibitory effect of c‐di‐GMP was partially suppressed by a mutant of YcgR, a protein with a PilZ domain and a binding site for c‐di‐GMP, suggesting that YcgR suppresses the rotational speed of the *E. coli* flagellar motor in response to the concentration of c‐di‐GMP. It has been reported that YcgR, activated by c‐di‐GMP, binds specifically to the rotator proteins FliM and FliG (Fang & Gomelsky, 2010; Paul et al., 2010) or the stator MotA (Boehm et al., 2010) (Figure 8). Biochemical approaches have confirmed the interaction between YcgR and MotA in the cytoplasmic region, with the C‐terminal domain of YcgR interacting with MotA. In contrast, the N‐terminal domain interacts with FliG (Hou et al., 2020). It is known that bacteria attach to the cell surface under starvation conditions to form multicellular populations. Under these conditions, high c‐di‐GMP concentration strengthens surface adhesion, favoring biofilm formation. Furthermore, deletion of *ycgR* reduces cell adhesion, suggesting that *ycgR*, a gene that suppresses flagellar motility, also plays a role in promoting biofilm formation (Fang & Gomelsky, 2010). *V. cholerae* has several PilZ domain proteins. It has been reported that c‐di‐GMP binds to PlzD, and the overexpression of PlzD in wild‐type strains on minimal medium reduces motility (Pratt et al., 2007). In *V. alginolyticus*, it has also been reported that motility is significantly inhibited when PlzD is expressed under oligotrophic conditions (Kojima et al., 2019). In *V. cholerae*, PlzC was found to promote biofilm formation only when c‐di‐GMP was at high concentrations. PlzB was found to promote biofilm formation regardless of the concentration of c‐di‐GMP (Conner et al., 2017). Both proteins are known to have a PilZ domain; however, it is not clear how they affect motility and biofilm formation. In particular, the mechanism of rotation inhibition of the Na^+^‐driven motor is similar to that of the H^+^‐driven motor described above.

## 9 FLAGELLAR GENES AND BIOFILM FORMATION

**FIGURE 9:**
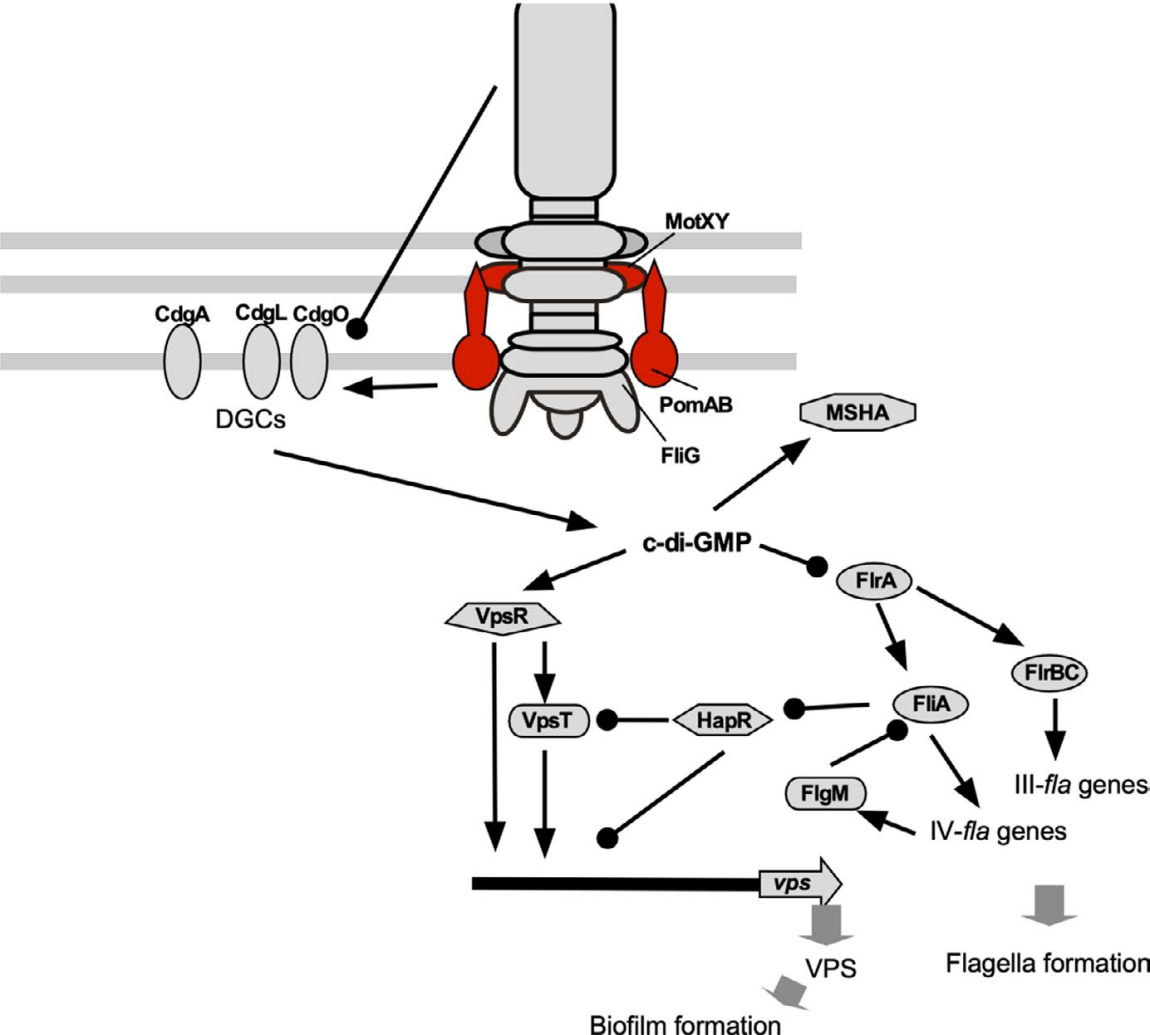
Model of signal transduction during flagellum‐dependent biofilm formation. The constitutive genes of the polar flagellum and biofilm‐forming genes are regulated by c‐di‐GMP signaling. The arrow ends represent positive regulation, and the black circle ends represent negative regulation. The figure was adapted from a previously published paper (Wu et al., 2020)

The relationship between flagellar gene deficiency and biofilm formation has been investigated in various studies. The biofilm formation response differs between serotype strains of O139 and O1(El Tol) in *V. cholerae*. It has been reported that strain O139 produces rugose colonies depending on the expression of the *vps* gene when the flagellar genes are deleted (Watnick et al., 2001). It has also been reported that the expression of CdgD DGC is regulated by the flagellar gene expression system (Syed et al., 2009). Thus, it seems that the flagellar gene regulatory system also regulates the expression of virulence factors of *V. cholerae* via CdgD. Deleting the *flaA* gene, a major flagellin gene in strain O139, causes the formation of rugose colonies. Furthermore, defects in *the pomA* or *motX* gene, a stator‐related gene of the flagellar motor, result in smooth colonies (Lauriano et al., 2004). This phenomenon is a result of changes in *vpsR* gene expression due to *pomA* or *motX* gene deletion. CdgA, CdgL, and CdgO were found to be involved in this phenomenon in a comprehensive study, in which 28 genes of DGCs in *V. cholerae* were mutated and their contribution to *vp* gene expression was investigated (Wu et al., 2020). It has been shown that CdgA alone has a strong effect on colony morphology and is involved in rugose colony formation (Beyhan et al., 2007). The *flaA* gene deletion increases intracellular c‐di‐GMP levels. These three proteins, CdgA, CdgL, and CdgO, seem to be responsible for increasing c‐di‐GMP levels. Since c‐di‐GMP regulates flagellar and *vps* genes' expression, this makes it a very complex response regulation (Figure 9).

## 10 CONCLUSION

Regulation by c‐di‐GMP is complex owing to the involvement of many factors. How changes in the concentration of c‐di‐GMP in flagellar mutants affect biofilm formation has not yet been revealed. However, recent studies have revealed that flagellar gene products interact with genes involved in the synthesis and degradation of c‐di‐GMP and regulate their activity. It has also been shown that the flagellar stator protein regulates the activity of membrane‐bound c‐di‐GMP synthesis and degradation proteins. In 2020, the structure of the flagellar stator, an energy‐conversion complex in the motor, was reported, and a major breakthrough for the mechanism of the force generation of flagellar motor is expected in this field. The role of viscosity and flagellar rotation as dynamometer and their involvement in gene expression will soon be clear in subsequent studies.

## CONFLICT OF INTEREST

The authors declare that there are no conflicts of interest.
